# Utility of Rapid Nasopharyngeal Swab for Respiratory Pathogens in the Diagnosis of Viral Infections in Children Hospitalized with Fever: A Prospective Validation Study to Improve Antibiotic Use

**DOI:** 10.3390/children11020225

**Published:** 2024-02-09

**Authors:** Danilo Buonsenso, Rosa Morello, Francesco Mariani, Bianca Mazzoli, Cristina De Rose, Ilaria Lazzareschi, Francesca Raffaelli, Rita Blandino, Maurizio Sanguinetti, Piero Valentini

**Affiliations:** 1 Department of Woman and Child Health and Public Health, Fondazione Policlinico Universitario A. Gemelli IRCCS, 00168 Rome, Italy; rosa.morello1@guest.policlinicogemelli.it (R.M.); francescomariani@omceoromapec.it (F.M.); cristina.derose@guest.policlinicogemelli.it (C.D.R.); ilaria.lazzareschi@policlinicogemelli.it (I.L.); piero.valentini@unicatt.it (P.V.); 2 Centro di Salute Globale, Università Cattolica del Sacro Cuore, 00168 Roma, Italy; 3 Department of Pediatrics, Catholic University of Rome, 00168 Rome, Italy; bianca.mazzoli@icatt.it (B.M.); rita.blandino01@icatt.it (R.B.); 4 Dipartimento di Scienze di Laboratorio e Infettivologiche, Fondazione Policlinico Universitario A. Gemelli IRCCS, 00168 Rome, Italy; francesca.raffaelli@policlinicogemelli.it (F.R.); maurizio.sanguinetti@policlinicogemelli.it (M.S.); 5 Dipartimento di Scienze Biotecnologiche di Base, Cliniche Intensivologiche e Perioperatorie, Sezione di Microbiologia, Università Cattolica del Sacro Cuore, 00168 Rome, Italy

**Keywords:** children, viral infections, bacterial infections, point-of-care test, fever, nasopharyngeal swab

## Abstract

**Introduction:** Fever is among the most common reason for medical assessment and antibiotic prescription in practice. The aim of this study was to evaluate positive and negative predictive values of rapid nasopharyngeal swabs for respiratory pathogens to discriminate viral from bacterial infections. **Methods:** We prospectively tested children with signs and/or symptoms of infections (e.g., fever, cough, wheezing, suspected urinary tract infection) admitted to a paediatric department. Following discharge, clinical phenotypes were assigned defining a cohort of children having probable/certain viral infection, probable/certain bacterial infection, other inflammatory conditions or healthy controls. **Results:** In this study, 190 children were enrolled (50.5% females, median age 30.5 (8–86) months). In total, 102 patients (53.7%) were affected by respiratory viral infections, 16 (8.4%) by bacterial infections, 29 (15.3%) were healthy controls and 43 (22.6%) were affected by another pathological condition manifested with fever. In total, 84.3% of patients classified as viral infection tested positive for viruses, compared with 18.8% of patients with bacterial infection (*p* < 0.001), 18.6% of patients with other condition (*p* < 0.001) and 17.2% of control patients (*p* < 0.001). The positive predictive value of NPSs in the diagnosis of viral infection was 88.6% and the negative predictive value was 75.0%. **Conclusion:** Our findings suggest that rapid NPS tests for respiratory viruses are a useful tool to confirm viral infections in children with fever and improve antibiotic use.

## 1. Introduction

Fever is one of the main reasons parents feel that their children need medical attention, either in outpatient settings or in the paediatric emergency department (PED) [1,2]. As such, fever is also one of the main reasons for doctors, including paediatricians, to prescribe antibiotic therapy [3]. Several studies have documented that one quarter to half of children evaluated in the PED for fever receive an antibiotic prescription [4,5,6], even when the analyses were restricted to children discharged by the PED and therefore representing the less severe spectrum of disease [7].

Antibiotic over-prescription for fever remains particularly high, despite the fact that it is well recognized that most children with fever have viral infections and do not benefit from antibiotics [8]. However, clinical prediction rules and biomarkers (both traditional ones like C-Reactive protein or new ones like procalcitonin) are useful but are not accurate enough to accurately distinguish viral from bacterial infections [9]. In addition, a strategy based on the routine performance of blood tests in all children with fever is not feasible for several reasons including costs, feasibility, time for results and invasiveness for children. Therefore, companies and researchers have focused their efforts on developing new tests able to detect respiratory viruses within 60–80 min, with the objective of supporting clinicians in the performance of a rapid and non-invasive diagnosis of viral infection and, therefore, limiting the use of antibiotics.

Such tests have received particular attention from clinicians and researchers as their implementation was expected to theoretically tackle antibiotic prescription, leading companies to develop rapid tests to be used directly at the patient’s bedside and at lower costs. However, a few randomized studies comparing children assessed with standard of care compared to a group of children tested with a nasopharyngeal swab (NPS) with results available within 70 min found a similar rate of antibiotic prescriptions in the two groups [10]. One of the main reasons hypothesized for a lack of effect of viral testing in reducing antibiotics is related to the possibility of having viral–bacterial coinfections, and therefore, a positive viral test would not certainly exclude a concomitant or occult bacterial infection [11,12,13,14,15]. In addition, a positive viral test may be considered as a colonizing virus, rather than the cause of the ongoing febrile illness [16]. However, such scenarios would be rare and would not justify the current high rate of antibiotic prescriptions. Importantly, many of these studies have not reported the definitive final diagnosis of these children; therefore, it remains uncertain how accurate viral testing would be in accurately identifying children with definite viral infection. Also, these studies have not included a control group of children tested but without having signs or symptoms of infection.

Given the theoretical important role of viral testing in reducing antibiotic prescriptions, and the lack of robust evidence in the literature, we implemented this prospective study aiming to assess the sensitivity and specificity of NPS testing for respiratory viral infections in children with signs and symptoms of infection, compared to a control group of children admitted for other reasons (e.g., elective surgery). In addition, compared to the available literature, we also tested the main caregiver in the hypothesis that viruses can easily spread within families and, therefore, having a confirmed viral infection in the caregiver would improve the accuracy of the test. We focused on admitted children in the attempt to understand the role of viral NPSs in the spectrum of severely ill feverish children.

## 2. Materials and Methods

The study took place in the Paediatric Department of Gemelli University Hospital in Rome, Italy, from 1 July 2022 to 31 December 2022. An NPS for respiratory viruses was taken from children admitted in the inpatient ward for signs and/or symptoms of infections (e.g., fever, cough, wheezing) and a control group of children admitted for elective surgery, along with the main caregiver that remained with the child during admission. In our Institution, children with signs and/or symptoms of infections are hospitalized if they need oxygen support, if the evaluating clinician defines the infection severe enough to be treated with intravenous antibiotics or if the clinical case is uncertain and deemed to receive further diagnostics to establish proper diagnosis (which would not be feasible to be complete in the setting of the Emergency Department). Patients were excluded if older than 18 years of age, NPS sampling was not possible (e.g., anatomical complications), the caregiver refused to be tested along with the child or did not sign the consent to participate. This study is part of a larger study investigating respiratory viruses in hospitalized children approved by the Ethics Committee of Gemelli University Hospital (ID 4990, protocol n 0020257/22). Signed informed consent was obtained by caregivers or, in the case of children older than 10 years of age, directly by them through an adapted consent, in line with local Ethical rules.

### 2.1. Data Collection 

For each child, the following variables were collected: date of birth, age, sex, reasons for hospitalisation, date of hospitalisation, infectious symptoms, date onset of symptoms, presence of specific signs of infections or clinical syndromes (fever, upper respiratory tract symptoms, pneumonia, urinary tract infections, gastroenteritis, suspected sepsis, febrile convulsions, bronchiolitis, mastoiditis, mononucleosis or hospitalisation for surgery) and concordance of symptoms with caregiver. We also collected the following hospitalisation variables: blood culture and urine culture, date of discharge, days of hospitalisation, antibiotic therapy (IV or oral) and antibiotic therapy at discharge if it is given. For the caregiver, we collected the same information regarding symptoms and their date of onset. 

For the caregiver, we collected the following information: date of birth, age, sex, reasons for hospitalisation, date of hospitalisation, infectious symptoms, date onset of symptoms, presence of specific signs of infections or clinical syndromes (fever, upper respiratory tract symptoms, pneumonia, urinary tract infections, gastroenteritis, suspected sepsis, febrile convulsions, bronchiolitis, mastoiditis, mononucleosis) and concordance of symptoms with the child).

### 2.2. Definition

Given the complexity in defining definite bacterial and viral infections in real settings, we used a recent, and more realistic, as it is more feasible in real world settings, definition proposed by the PERFORM consortium (Personalised Risk assessment in febrile children to optimise Real-life Management across the European Union), which proposes a spectrum of conditions leading to fever rather than a simple dichotomous classification (viral vs. bacterial) [5]. Briefly, the proposed new algorithm provides a framework for phenotyping children with probable infections according to the certainty of the diagnosis of either bacterial or viral categories. Following discharge, clinical phenotypes were assigned by an expert in paediatric infectious diseases and two senior residents after a review of all available clinical and laboratory data including biochemistry, haematology, radiology and microbiology, therefore reflecting routine clinical practice (where, in most cases, invasive procedures to perform microbiological gold-standard tests in sterile fluids are not ethically feasible). This process was performed by a team of reviewers that were unaware of the NPS results at the time of performance, and unaware of the study goals, but were aware of the final swab results. In light of all the clinical information, the team of reviewers classified children as having probable/certain viral infection, probable/certain bacterial infection, other inflammatory conditions or as healthy controls. Examples of allocation of cases in definite/probable bacterial/viral infections are reported in the supplementary material of the reference study [5]. For example, a child with a clinical diagnosis of bronchiolitis and positive NPS for viruses is considered a case of definite viral infections, while a child with febrile seizures (simple or complex) with a positive NPS for viruses but negative results on cerebrospinal fluid, exclusion of bacterial infections, normal or low inflammatory markers or who improved without antibiotics is considered as probable viral infection. Both these examples end in the group of children classified as “viral infection”. Similarly, children with microbiologically confirmed bacterial infections in clinically relevant specimens and clinical scenarios (urinary tract infection, sepsis, empyema or meningitis) are included in the “bacterial group”, as well a child with lobar pneumonia and high inflammatory markers without bacterial cultures from sterile samples and who improved with antibiotics, even if a positive NPS for viruses was obtained (probable bacterial case).

Children with inflammatory conditions (e.g., Kawasaki disease, autoimmune diseases, fever of unknown origin without a final aetiological diagnosis, Crohn’s disease, Ulcerative colitis) were classified as “other”. Last, healthy children admitted for planned surgery (e.g., children admitted for a nasal or wrist trauma or congenital hip dysplasia with need of surgery) were enrolled as “healthy controls”. 

### 2.3. Outcome 

The primary outcome was the positive predictive value (PPV) and negative predictive value (NPV) of PSP for confirmed/probable viral infection.

The secondary outcomes were the

1. Number of children with an antibiotic prescription in the group of children with positive viral NPS test;
2. Evaluation of the accuracy of NPS test in recognizing confirmed/probable viral infections when the concordance between child and caregiver test was considered;
3. Overall concordance between child and caregiver of the NPS tests and according to subgroups of children with specific infections.

### 2.4. NPS Testing

As we previously reported (REF) [17], the QIAstat-Dx Respiratory SARS-CoV-2 Panel is a qualitative test intended for analysing nasopharyngeal swab (NPS) samples from patients suspected of respiratory infection for the presence of viral or bacterial nucleic acids (https://www.qiagen.com/zh-us/products/diagnostics-and-clinical-research/infectious-disease/qiastat-dx-syndromic-testing/qiastat-dx-eua-us, accessed on 1 October 2023). The QIAstat-Dx Respiratory SARS-CoV-2 Panel is able to accept both dry swabs and transport medium liquid samples. The assay is designed for use with the QIAstat-Dx Analyzer 1.0 and QIAstat-Dx Rise for integrated nucleic acid extraction and multiplex real-time RT-PCR detection [18].

The QIAstat-Dx Respiratory SARS-CoV-2 Panel detects and differentiates* SARS-CoV-2, Influenza A, Influenza A subtype H1N1/2009, Influenza A subtype H1, Influenza A subtype H3, Influenza B, Coronavirus 229E, Coronavirus HKU1, Coronavirus NL63, Coronavirus OC43, Parainfluenza virus 1, Parainfluenza virus 2, Parainfluenza virus 3, Parainfluenza virus 4, Respiratory Syncytial virus A/B, human Metapneumovirus A/B, Adenovirus, Bocavirus, Rhinovirus/Enterovirus, *Mycoplasma pneumoniae*, *Chlamydophila pneumoniae, Legionella pneumophila* and *Bordetella pertussis*. The test result was defined “concordant” when the same virus was found in both the patient and the caregiver.

### 2.5. Statistical Analyses

For continuous variables, we used the Kolmogorov–Smirnov test to evaluate whether the distribution was normal or not. Categorical variables were reported as count and percentage. Continuous variables with normal distribution were reported as mean with standard deviation; data with skewed distribution were reported as median and interquartile range (IQR 25–75%). Statistical comparisons between groups were obtained by Chi-squared tests or Fisher’s exact tests for categorical variables and by the Mann–Whitney U-test or T-test for continuous variables. For proportion comparison, Bonferroni correction was applied for multiple analysis. The Kruskal–Wallis test was used for comparison of continuous variables between more than 2 groups. 

The positive predictive value of the test was assessed according to the following formula: true positive/(true positive + false positive). Those values are reported with a 95% confidence interval. 

The negative predictive value was assessed according to the following formula: true negative/(true negative + false negative). *p* value < 0.05 was considered statistically significant.

Statistical analysis was performed using IBM SPSS Statistics 25.0 software (IBM Corporation, Armonk, NY, USA).

## 3. Results

### 3.1. Study Population

A total of 190 couples of patient/caregivers admitted to the paediatric ward of the Policlinico Agostino Gemelli University Hospital in Rome, in the period between July and December 2022, were enrolled. Among the 190 enrolled children (96 females, 50.5%), 102 patients (53.7%) were affected by respiratory viral infections, 16 (8.4%) by bacterial infections, 29 (15.3%) were healthy controls and 43 (22.6%) were affected by another pathological condition manifested with fever. The median age was 30.5 (8–86) months. The result of the nasal swab showed concordance (either both positive, or both negative) between child/caregiver in 130 cases (68.4%), while in 38 child/caregiver couples (20%), symptoms were present in both the child and the adult. The details of the frequencies of those characteristics in the different diagnostic subgroups are reported in Table 1. 

The main symptoms, infectious syndromes diagnosed, results of cultures and use of antibiotics in the cohort are reported in Table 2. Overall, 67 children (35.3%) started antibiotics on admission with a median duration of 5 (3–8) days, despite the fact that 16 were eventually recognized as having a bacterial infection and only 13 children (5.9%) out of 34 patients who performed blood culture tests tested positive and 11 (34.4%) out of 32 children who performed urine culture tests had a clinically relevant culture positive for bacterial infection.

Out of 102 patients with respiratory viral infection, 23 children (22.5%) received antibiotics.

### 3.2. NPS in Children Classified in the Different Diagnostic Groups

Proportion of patients who tested positive in the different diagnostics subgroups and a comparison between the proportion of positive patients between those affected by respiratory viral infection and those affected by all the other conditions are reported in Table 3. A statistically significant difference was observed when we compared data of the entire population; in detail, 84.3% of patients classified with viral infection tested positive, compared with 18.8% of patients with bacterial infection (*p* < 0.001), 18.6% of patients with another condition (*p* < 0.001) and with 17.2% of control patients (*p* < 0.001). In Table 3, we also reported the presence of positive NPSs for viruses in each diagnostic group according to a child/caregiver concordant or discordant test. Having a child/caregiver couple with a concordant NPS positive for viruses further reduced the probability that the feverish child would have a bacterial infection.

On the left side of the table, the values are reported as N of patient who tested positive/n of patients tested and percentage. The *p* values are from the chi square test used for proportion comparison and are adjusted according to Bonferroni correction for multiple analysis.

For the entire population, the positive predictive value of NPSs in the diagnosis of viral infection was 88.6% (95% CI 80.8–93.5) and the negative predictive value was 75.0% (95% CI 63.2–84.0). In the population of patients whose nasal swab was concordant with the caregiver’s one, the positive predictive value was 89.6% (95% CI 77.8–95.5), while the negative predictive value was 76.7% (95% CI 64.6–85.6). In the population of patients whose nasal swab was non-concordant with the caregiver’s one, the positive predictive value was 87.7% (95% CI 75.8–94.3), while the negative predictive value was 50.0% (95% CI 8.9–91.1) (Table 4).

To better evaluate the positive predictive value and negative predictive value, we removed controls from this analysis.

The viral load was evaluated reporting the cycles of the nasal swab result. The median value of cycles in the viral infected patients was 28.25 (24.2–32.02), the median value in the bacterial infection patients was 29.8, the median value of the controls was 31.8 (27.7–34.4) and the median value for patients affected by other conditions was 30.9 (24.9–33.0). No statistically significant difference was observed in the comparison of those values (*p* = 0.41).

We further evaluated the frequency of a positive NPS for viruses in both the child and caregiver (concordant result) or not. We found that all viruses, except Parainfluenza Virus 3, might be found in both children and the caregiver and at similar viral loads, suggesting that viruses traditionally thought to colonize in children (like Respiratory Syncytial Virus–RSV) can also colonize in adults. However, the probability of concordant or discordant results for a specific virus was non-statistically significant (Table 5).

### 3.3. Analysis of Children with Clinically Defined Bacterial Infection and NPS Positive for Viruses

A subgroup analysis was performed to evaluate the characteristics of children affected by bacterial infection. In particular, three children diagnosed with bacterial infections also had an NPS positive for viruses (Table 6). In Table 7, the characteristics of the three patients affected by bacterial infection who tested positive with NPS are collected. 

Of them, two had a microbiologically confirmed urinary tract infection (the viral isolates were considered as colonization), and one child had a diagnosis of mastoiditis with high inflammatory markers (in this case, the virus may have been a colonizer or a contributing pathogen to drive the initial upper airway infection and the subsequent complication). 

## 4. Discussion

In this study, we prospectively evaluated a rapid NPS for respiratory pathogens in children admitted with fever and their caregivers. Overall, we found that children eventually classified has having a viral infection had a significantly higher probability of having a positive virus in the NPS compared to children with bacterial infection, other inflammatory conditions or control groups (*p* < 0.001). Overall, the positive predictive value of NPSs for viruses in the diagnosis of viral infection was 88.6% and the negative predictive value was 75.0%. All together, these findings suggest that rapid NPS tests for respiratory viruses are a useful tool, although not perfect, to suspect viral infections in children with fever.

Nevertheless, studies published before our analyses have found contradictory results about the potential role of viral NPSs in reducing antibiotic prescriptions. A systematic review (due to high heterogeneity, an overall meta-analysis was not possible) published in 2019 and including 23 studies did not find, overall, difference in the proportion of patients receiving antibiotics between those with positive versus negative test results, even in subgroup analyses by age, respiratory virus test type and viral target. However, stratification by study design revealed that viral NPS testing decreased antibiotic use in prospective cohort studies (odds ratio 0.58; 95% confidence interval: 0.45–0.75) [19]. Despite the fact that it is well known that most children with fever have viral infections, it is possible that clinicians keep prescribing antibiotics in children with positive viral tests for several reasons, including behavioural ones and fear of parents/legal repercussions in a case of misdiagnosis [7]. In fact, at least theoretically, it would be expected that children with fever and positive viral tests would be initially considered as having a viral infection until proved otherwise and would receive antibiotics only in select cases of strong clinical suspicion of bacterial infection and other diagnostics further supporting a possible ongoing bacterial infection.

In our study, we have found that children classified as having a viral infection have statistically significant higher rates of positive NPSs compared to controls and children with possible bacterial or other inflammatory conditions. Our findings suggest that children with a viral-positive NPS and a coherent clinical presentation may be considered as having a viral infection until proven otherwise and, therefore, routine antibiotic prescriptions in such cohorts of children should be discouraged, unless children appear severely ill. Although, recent studies have found that clinicians do not reduce antibiotic prescriptions when they are aware of a rapid viral positive test [19]. We believe that positive NPSs are accurate enough for predicting real viral infections in clinically stable children, and in these cases, routine antibiotic prescriptions are clinically unjustified. Of note, the enrolled cohort is representative of the target for the NPS, and rates of bacterial and viral infections are in line with larger European cohorts (in both cases, around only 10–15% of children with fever had a bacterial infection), including the relatively high proportion of children with other inflammatory conditions that initially presented with fever [20]. It is possible that other reasons lead clinicians to frequently prescribe antibiotics in children with positive viral swabs, including psychological factors related to legal consequences in case of missed bacterial infections. However, given what is known by historical epidemiologic data (viral infections are the commonest cause of fever in children, and bacterial–viral coinfections are relatively rare), we believe that our study further reinforces that children with positive viral swabs with a clinically coherent presentation should be considered as infected by viruses until proven otherwise. In agreement with our findings, in a recent prospective study performed in Denmark, using a point-of-care NPS test, the availability of fast results from bedside analysis significantly changed the prescribed antibiotic treatment to non-antibiotic treatment in 46% (36–56%) of the children and the reverse in 2% (1–8%) [21]. 

**Figure 1:**
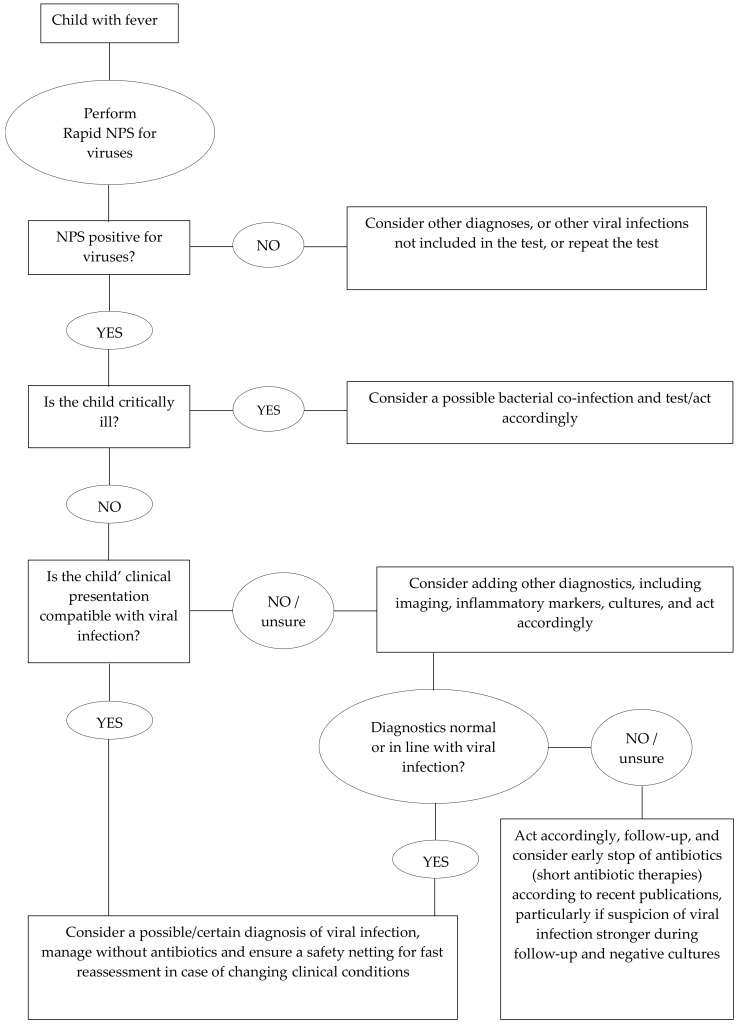
Flow chart showing a possible approach to using nasopharyngeal swabs in children with fever.

It is important to highlight that three children (representing 18.8% of those with bacterial infections) also tested positive for viruses, highlighting what is already known about a good, but not perfect, PPV of viral tests to rule-out bacterial infection. However, this is not surprising since the complexity of medicine and clinical presentations have never led to the development of single diagnostics that have 100% sensitivity and specificity for a specific condition. Rather, the diagnostic process is a complex activity that requires the elaboration of several parts of information, from clinical history and examination to different steps of diagnostics. In this context, rapid NPSs need to be considered as one of the tools that clinicians have to diagnose a viral (or bacterial) infection, in a process that needs to be personalized based on each child’s presentation. Given their good positive and negative predictive values, in combination with rapid results and low invasiveness, rapid NPSs can be used as a useful initial assessment tool, which may require, or not, further approaches to confirm a clinical suspicion according to the specific scenario, as suggested in Figure 1. Importantly, no single test will probably ever prevent a child diagnosed with a mild, viral infection, worsening in the following days and coming back with a serious bacterial infection. The reasons are multifactorial (e.g., viral infections can facilitate subsequent bacterial infections, even days to weeks after the initial infection), but clinicians and the public should be aware that these scenarios only rarely refer to wrong medical decisions but instead to new events. As mentioned in a famous emergency medicine editorial, “sick children look sick”, the child returning with worsening clinical conditions is, in most cases, simply unpredictable.

In this study, we also tested the caregiver taking care of the child during admission and we found that in 68.4% of cases both the child and the caregiver had the same results. We tested both the child and caregivers to evaluate two hypotheses: first, if both the child/caregiver had the same virus, this finding would strengthen the suspicion that the isolated virus was the cause of the infectious illness, rather than a simple coloniser. Interestingly, when we considered a child/caregiver concordant positive viral swab, the probability of misinterpreting a bacterial infection as viral was even lower, suggesting that when both the child and the caregiver carry the same virus, it is probable that there is an actively replicating virus that is causing the ongoing infectious disease. Secondly, on an epidemiologic perspective, children have been historically considered the main transmitters of common respiratory viruses like Respiratory Syncytial Virus or influenza [22]. However, the recent pandemic has shown us that many of the historical paradigms about viruses’ transmission should be challenged. During the first years of the pandemic, when several non-pharmacological measures were implemented, particularly in adults, all viruses except COVID-19 disappeared. Later, as soon restrictions were lifted, a significant rebound of viral circulation was noted, even in those countries that did not establish paediatric restrictions like in northern Europe, leading some authors to speculate that adults may play a role in widening the circulation of common viruses on a larger scale, being vectors in a similar way to children [23]. Adults, in fact, have much more encounters and travel globally compared to children. Interestingly, we found all common viruses also in adult samples, including rhinovirus/enterovirus, RSV, adenovirus and others. In addition, viral loads in our cohorts of adults and children were similar, suggesting that, at least theoretically, adults bear similar viral loads as their children and have the potential to contribute, by amplifying, to the spread of viruses globally.

Our study is not without limitations. First, a definitive microbiological confirmation in paediatric practice is challenging, and we cannot exclude that some of the children included in the “bacterial infection” group had actually viral infections. However, this is an intrinsic limitation for aetiological studies in paediatric practice and will remain difficult to be overcome until new specific biomarkers of bacterial infections are implemented. In addition, although we included a relatively large sample of children, the number of isolates for each virus was not large enough to allow more statistical comparisons for each subgroup. Third, the study was performed in Rome from July 2022 to December 2022, therefore including both a period of low and high viral circulation but missing the peak period of RSV and Influenza virus circulation (disrupted by previous pandemic restrictions); therefore, this is a limitation that requires the replication of similar studies in periods of regular viral circulation. The numbers were not large enough to perform comparisons of the PPV/NPV of NPSs during these different epidemiological periods. Fourth, since January, Italy (including our centre) experienced a surge of empyema like other European countries (probably with Group A Streptococcus playing a relevant role). However, our study was concluded in December. Therefore, we cannot exclude that during periods of higher circulation of severe bacterial infections, the PPV/NPV of viral NPSs may differ. Last, in our opinion, a common limiting factor of studies evaluating the role of NPSs in differentiating viral from bacterial infections is in implementing scoring systems that not only evaluate the NPS but also clinical data, laboratory findings and “clinician gut feelings”. In this study, we have not considered this information. Last, during the study period we did not isolate any of the bacterial pathogens included in the test we used; therefore, we could not determine the PPV and NPV of a positive test for bacteria.

## 5. Conclusions

In conclusion, we found that rapid NPSs have good positive and negative predictive values to support a clinical diagnosis of viral infection in most children with fever. However, such tests should always be used along with other clinical information. Future studies should implement multiparametric models that, combining several forms of information, may help to provide a more accurate and personalized classification of children with fever and therefore improve the way we diagnose and manage our children.

## Figures and Tables

**Table 1:** Characteristics of study population.

	Study Population (N = 190)	Respiratory Viral (N = 102)	Bacterial (N = 16)	Other (N = 43)	Control (N = 29)
**Female**	96 (50.5)	55 (53.9)	7 (43.8)	24 (55.8)	10 (34.5)
**Age (months), *median IQR***	30.5 (8–86)	15 (6–43)	39 (14–141)	39 (9–106)	146 (60–172)
**Nasal swab result concordant** **(all results, including negative tests)**	130 (68.4)	57 (55.9)	14 (87.5)	37 (86.0)	22 (75.9)
**Symptoms concordant**	38 (20)	21 (20.6)	1 (6.3%)	4 (9.3)	12 (41.4)

**Table 2:** Clinical, microbiological sampling and therapy characteristics of the entire population.

	Study Population (N = 190)
**Days of hospitalization, *median IQR***	4 (0–7)
**NPS positive**	102 (53.7)
**Infective symptoms**	161 (84.2)
**Fever**	96 (50.5)
**Rhinitis**	99 (52.1)
**Pneumonia**	11 (5.8)
**Urinary tract infection**	4 (2.1)
**Gastroenteritis**	5 (2.6)
**Suspected sepsis**	1 (0.5)
**Febrile seizures**	11 (5.8)
**Bronchiolitis**	25 (13.2)
**Otomastoiditis**	1 (0.5)
**EBV infection**	2 (1.1)
**Surgery**	14 (7.4)
Blood culture positive *	2 (5.9)
Urine culture positive **	11 (34.4)
**Antibiotic therapy performed**	67 (35.3)
**Days of antibiotic therapy, *median IQR***	5 (3–8)
**Intravenous antibiotic therapy**	56 (29.5)
**Days of intravenous antibiotic therapy, *median IQR***	4 (2–7)

* Blood cultures performed for 34 pts. ** Urine cultures performed for 32 pts.

**Table 3:** Proportion of patients tested positive with NPS in the different diagnostic classes.

	Respiratory Viral Infection	Bacterial Infection	Other Condition	Controls	Respiratory Viral Infection vs		
					Controls	Bacterial Infection	Other Condition
**NPS (entire population)**	**86/102** **(84.3%)**	**3/16** **(18.8%)**	**8/43** **(18.6%)**	5/29 (17.2%)	<0.001	<0.001	<0.001
**NPS (concord population)**	43/57 (75.4%)	1/14 (7.1%)	4/37 (10.8%)	0/22 (0%)	-	<0.001	<0.001
**NPS (non-concord population)**	43/45 (95.6%)	2/2 (100%)	4/6 (66.7%)	5/7 (71.4%)	NS	-	0.04

**Table 4:** Positive predictive value and negative predictive value of NPS, with their confidence intervals, in the diagnosis of viral infection.

	PPV	NPV
**NPS (entire population)**	88.6 (80.8–93.5)	75.0 (63.2–84.0)
**NPS (concord population)**	89.6 (77.8–95.5)	76.7 (64.6–85.6)
**NPS (non-concord population)**	87.7 (75.8–94.3)	50.0 (8.9–91.1)

PPV: positive predictive value; NPV: negative predictive value; NPS: nasopharyngeal swabs.

**Table 5:** Comparison between the proportion of each virus detected in the nasal swab for discord NPS group and in nasal swab for concord NPS group.

	Nasal Swab Discord (N = 54)	Nasal SWAB Concord (N = 48)	*p*
**Rhino/Enterovirus**	34 (58.6)	24 (41.4)	NS
**Adenovirus**	2 (50.0)	2 (50.0)	NS
**SARS-CoV-2**	2 (33.3)	4 (66.7)	NS
**Parainfluenza 3**	3 (100.0)	0 (0)	NS
**Parainfluenza 4**	2 (66.7)	1 (33.3)	NS
**Parainfluenza 2**	1 (50.0)	1 (50.0)	NS
**Bocavirus**	1 (100.0)	0 (0)	NS
**RSV**	8 (42.1)	11 (57.1)	NS
**Influenza AH3**	1 (25.0)	3 (75.0)	NS
**Coronavirus OC43**	0 (0)	1 (100.0)	NS
**Influenza B**	0 (0)	1 (100.0)	NS

The values are reported as N and percentage of the raw *p* values from *z* test for proportion comparison.

**Table 6:** Characteristics of children classified as bacterial group.

	Negative Viral Swab (N = 13)	Positive Viral SWAB (N = 3)	Non-Concordant Viral Swab (N = 2)	Concordant Viral Swab (N = 14)
**Infective symptoms**	12 (92.3)	3 (100)	2 (100)	13 (92.9)
**Fever**	11 (84.6)	2 (66.7)	1 (50)	12 (85.7)
**Rhinitis**	2 (15.4)	1 (33.3)	0 (0)	3 (21.4)
**Pneumonia**	4 (30.8)	0 (0)	0 (0)	4 (28.6)
**Urinary tract infection**	1 (7.7)	1 (33.3)	1 (50)	1 (7.1)
**Gastroenteritis**	0 (0)	0 (0)	0 (0)	0 (0)
**Suspected sepsis**	1 (7.7)	0 (0)	0 (0)	1 (7.1)
**Febrile seizures**	0 (0)	0 (0)	0 (0)	0 (0)
**Bronchiolitis**	0 (0)	0 (0)	0 (0)	0 (0)
**Otomastoiditis**	0 (0)	1 (33.3)	1 (50)	0 (0)
**EBV infection**	0 (0)	0 (0)	0 (0)	0 (0)

**Table 7:** Details of the three children with bacterial infections and positive NPS.

	Patient 1	Patient 2	Patient 3
**Age**	2 years	17 days	3 years
**Sex**	M	M	M
**Comorbidities**	No	No	No
**Reason for admission**	UTI, Suspect urosepsis	Fever, Suspect urosepsis	Otomastoiditis
**Fever**	No	Yes	Yes
**Virus detected**	SARS-CoV2	Rhino/enterovirus	Rhino/enterovirus
**UTI**	Yes	Yes	No
**CAP**	No	No	No
**Bronchiolitis**	No	No	No
**Upper respiratory tract symptoms**	No	Yes	No
**Gastroenteritis**	No	No	No
**Febrile convulsions**	No	No	No
**Otomastoiditis**	No	No	Yes
**Mononucleosis**	No	No	No
**Hospitalization for surgery**	No	No	No
**Parent symptoms concordance**	No	No	No
**Parent swab concordance**	No	Yes	No
**Blood culture**	Negative	Negative	/
**Urine culture**	Positive (*E. coli*)	Positive (*E. coli*)	/
**PCR**	129.2 mg/L	166 mg/L	313.6 mg/L
**PCT**	5.83 ng/mL	1.18 ng/mL	/
**Final diagnosis**	UTI	UTI	Otomastoiditis
**Duration of antibiotic therapy (days)**	12	19	10

## Data Availability

Due to local laws on personal data, the data cannot be shared publicly. To request these data, please contact the corresponding author for more information.
